# The Potential of Mesenchymal Stem Cell-Derived Exosomes to Treat Diabetes Mellitus

**DOI:** 10.3390/biomimetics10010049

**Published:** 2025-01-14

**Authors:** Ju-El Kim, Jong-Won Lee, Gi Doo Cha, Jeong-Kee Yoon

**Affiliations:** Department of Systems Biotechnology, Chung-Ang University, Anseong-si 17546, Gyeonggi-do, Republic of Koreacgidoo@cau.ac.kr (G.D.C.)

**Keywords:** diabetes mellitus, mesenchymal stem cells, exosomes, tissue regeneration

## Abstract

Diabetes mellitus (DM) is a fatal metabolic disease characterized by persistent hyperglycemia. In recent studies, mesenchymal stem cell (MSC)-derived exosomes, which are being investigated clinically as a cell-free therapy for various diseases, have gained attention due to their biomimetic properties that closely resemble natural cellular communication systems. These MSC-derived exosomes inherit the regenerative and protective effects from MSCs, inducing pancreatic β-cell proliferation and inhibiting apoptosis, as well as ameliorating insulin resistance by suppressing the release of various inflammatory cytokines. Consequently, MSC-derived exosomes have attracted attention as a novel treatment for DM as an alternative to stem cell therapy. In this review, we will introduce the potential of MSC-derived exosomes for the treatment of DM by discussing the studies that have used MSC-derived exosomes to treat DM, which have shown therapeutic effects in both type 1 and type 2 DM.

## 1. Introduction

Diabetes mellitus (DM) is a metabolic syndrome that affects an estimated 537 million adults aged 20–79 years (10.5% of all adults within this age range) globally [1,2,3,4], with the number of affected individuals showing a steadily increasing trend over recent years [1,2,5]. The persistently high blood glucose levels caused by DM can lead to various complications that affect a patient’s quality of life [1,6,7,8,9]. However, no widely implemented treatments capable of addressing the pathophysiological mechanisms of diabetes have been developed to date, highlighting the urgent need for research into such therapeutic approaches [6].

DM is mainly categorized into type 1 DM (T1DM) and type 2 DM (T2DM) [1,2,10]. T1DM is an autoimmune disease in which the β-cells in the islets of Langerhans in the pancreas are destroyed, leading to a loss of insulin production [11,12,13]. T2DM, which accounts for approximately 90% of diabetes cases, is characterized by insulin resistance in peripheral tissues, including the liver and skeletal muscles, and is often associated with obesity [14,15,16]. Conventional therapies for DM include oral hypoglycemic agents (OHAs), insulin injections, stem cell therapy, and pancreas transplantation [8,11,14]. Insulin injections are effective in temporarily alleviating symptoms, and oral hypoglycemic agents (OHAs) have been reported to transiently improve insulin resistance and enhance insulin production. However, these approaches cannot be considered fundamental treatments. Furthermore, stem cell therapy and pancreas transplantation can restore pancreatic function but are accompanied by various side effects (e.g., transmission of infections, immune compatibility), underscoring the need for the development of new therapeutic approaches [6,7,8,11,14].

Mesenchymal stem cells (MSCs) are non-hematopoietic stem cells derived from various tissues such as bone marrow, adipose tissue, umbilical cord, Wharton’s jelly, and placenta [17,18,19]. MSCs have multipotency and immunomodulatory and tissue regenerative effects. The MSCs have been demonstrated and are being investigated for the treatment of neurodegenerative and inflammatory diseases [17,18,20,21]. Recent studies have shown that co-culture or in vivo infusion of MSCs can ameliorate DM symptoms and that stem cell-based therapies can replace or regenerate destroyed β-cells [1,8,11,14]. However, repeated failures in clinical practice have been reported due to their low engraftment rate, therapeutic efficacy, and the difficulty of managing unexpected side effects (e.g., transmission of infections, immune compatibility, and embolism formation) [8,14].

Exosomes are spherical-shaped membranous extracellular vesicles (EVs) with a particle size ranging from 30 to 150 nm [1,22]. Secreted by most types of cells, exosomes have received considerable attention as a new alternative to stem cell therapy for DM because of their biomimetic carrier capacity to load various bioactive molecules, such as proteins, carbohydrates, and lipid membranes inherited from the parent cells, as well as their role in intercellular communication through paracrine or endocrine signaling [8,23,24,25]. This property allows exosomes to mimic the physiological effects of the parent cells, such as tissue regenerative and immunomodulatory effects on the target cells [23,24]. By inherently mimicking the signaling and functional attributes of their parent cells, MSC-derived exosomes are a promising approach for the treatment of DM, leveraging their characteristics to achieve efficient therapeutic outcomes.

In this review, we will provide an overview of the pathogenesis of DM and the advantages and limitations of existing therapeutic approaches, the use of MSCs for the treatment of DM, and explore the applications of MSC-derived exosomes in tissue regeneration. We will then discuss in detail the role of exosomes in DM and what research is currently underway. In T1DM, we will examine studies on the relationship between exosomes and immune regulation and β-cell regeneration, and in T2DM, we will explore studies investigating the role of exosomes in insulin resistance. Finally, we propose improvements that need to be addressed for MSC-derived exosome therapy to be effectively implemented in clinical practice.

## 2. DM: Pathophysiology and Conventional Therapy

While the exact cause of DM remains unknown, various hypotheses have been proposed, and experiments have been conducted to investigate its cause. Alongside these efforts, various therapeutic agents are currently being used to treat DM. The primary approach to managing DM has traditionally been exogenous insulin injection. However, in recent years, various therapeutic strategies aimed at addressing the underlying causes of DM have been explored and developed.

### 2.1. T1DM

T1DM is an autoimmune disease in which the β-cells in the islets of Langerhans are destroyed by an abnormal response of the immune system, resulting in the loss of insulin secretion [11,12,13]. The antibody against the 65 kDa isoform of glutamic acid decarboxylase, an enzyme expressed in β-cells, is one of the most common markers appearing during the early stages of T1DM [12,26,27,28]. When autoantibodies recognize these β-cell antigens, antigen-presenting cells subsequently activate the CD4+ and CD8+ T cells, initiating an autoimmune response against β-cells [12,26,27]. This autoimmune response leads to the gradual destruction of β-cells, ultimately causing the loss of insulin secretion function [11,12,13].

Surgical procedures such as islet transplantation and pancreas transplantation are performed to restore the damaged β-cells and insulin secretion in individuals with T1DM [29,30,31]. Pancreas transplantation offers the advantage of enabling approximately 75% of recipients to achieve sustained insulin independence. However, the necessity for long-term immunosuppressive therapy poses risks of associated complications. Islet transplantation is less invasive compared to pancreas transplantation and is associated with a lower incidence of complications [32]. Nevertheless, its limitations include a relatively low insulin independence rate of approximately 25% at five years post-transplantation and the restricted availability of cadaveric islets [11,31]. While stem cell-based β-cell replacement has been highlighted as a potential solution to address the limited availability of cadaveric islets, a solution to post-transplant rejection is still required [11,29,33]. Given that T1DM is an autoimmune disease, immunotherapies that modulate T cells and pro-inflammatory cytokines have been actively investigated over recent years [34,35]. However, these options also face technical challenges in translating preclinical animal data to humans [35].

### 2.2. T2DM

T2DM is fundamentally caused by insulin resistance, which is characterized by an impaired response of insulin-responsive cells (e.g., myocytes, adipocytes, hepatocytes) to circulating insulin levels, and is the fundamental cause of T2DM [14,36,37,38]. Insulin-sensitive tissues, such as muscles, adipose tissues, and the liver, are critical to metabolic processes, and insulin resistance within these tissues is a crucial factor for the progression to T2DM [14,36,37]. The interaction between the insulin receptor and insulin facilitates the GLUT4 translocation to the plasma membrane, enabling glucose uptake [37,39,40]. Mutations in signaling pathway molecules associated with this process, such as insulin receptor substrate (IRS)-1, IRS-2, and phosphoinositide 3-kinase, contribute to the development of insulin resistance [37,39]. In particular, hypertrophied adipose tissue resulting from obesity, along with adipose tissue-resident immune cells, increases circulating pro-inflammatory cytokines, inducing chronic inflammation that impairs insulin signaling pathways and exacerbates insulin resistance [7]. Furthermore, systemic metabolic disorders arising from insulin resistance lead to glucotoxicity and lipotoxicity in pancreatic β-islets, accelerating β-cell damage, promoting apoptosis, and worsening the progression of T2DM progression [41,42,43].

OHAs are used to control blood glucose levels in T2DM and are divided into several classes according to their mechanism of glycemic control [44]. For instance, metformin has the effect of increasing insulin sensitivity in peripheral tissues, whereas sulfonylureas have the mechanism of overcoming insulin resistance by stimulating insulin secretion from β-cells and increasing blood insulin levels [8,45,46]. All OHAs show excellent glycemic control; nevertheless, depending on the mechanism of glycemic control, they are associated with side effects such as hypoglycemia, hypo-leukocytosis, hemolytic anemia, gastrointestinal response, and increased risk of cardiovascular disease [8]. Bariatric surgery, used to treat T2DM in obese patients, involves modifying the gastrointestinal tract to reduce calorie intake and improve insulin sensitivity. It offers benefits like rapid glycemic control and high remission rates but carries risks such as nutritional deficiencies and surgical complications [44].

The existing methods of therapy do not solve the problem of DM, and the search for new methods of therapy is an urgent task of modern biology and medicine. In particular, exosomes reportedly improve insulin resistance and regenerate β-cells, making exosome-based DM treatment a new alternative [20].

## 3. MSC Therapy in DM

The destruction of pancreatic islet β-cells due to autoimmune responses, along with pancreatic β-cell dysfunction and insulin resistance, are key markers of T1DM and T2DM, respectively [47,48]. Existing treatments, such as oral antidiabetic drugs, antihyperglycemic agents, and exogenous insulin injections, temporarily improve insulin sensitivity in tissues or mitigate hyperglycemia-related symptoms. Nevertheless, these treatments do not reverse disease progression or restore cellular dysfunction, nor do they accurately mimic the physiological functions of endogenous insulin [49,50]. Moreover, in certain cases, these therapies have been reported to show ineffectiveness, pose an increased risk of hypoglycemia, and cause various side effects [51,52,53].

MSCs have demonstrated significant potential in diabetes management due to their inherent immunomodulatory and regenerative capabilities. Preclinical studies have shown that MSC therapy can improve insulin sensitivity, promote the regeneration of pancreatic β-cells, and reduce systemic inflammation [54,55,56]. MSCs enhance insulin sensitivity by promoting increases in protein kinase B, which is essential for insulin signaling and glucose uptake, and by inducing higher levels of GLUT4 expression [54,57]. In addition, MSC administration drives macrophage polarization toward the anti-inflammatory M2 phenotype and reduces pro-inflammatory cytokines, such as IL-6 and TNF-α [55]. Collectively, these effects regulate insulin secretion and modulate insulin resistance, positioning MSCs as a therapeutic approach for diabetes management. Furthermore, MSCs secrete a variety of bioactive substances, including cytokines, chemokines, growth factors, and exosomes, which significantly promote the regeneration and survival of pancreatic β-cells, thereby mediating therapeutic effects in diabetes [58,59]. MSCs derived from bone marrow (BM) and umbilical cord sources have been particularly effective in reducing apoptosis in pancreatic β-cells and enhancing the survival of islet cells [60,61]. Moreover, MSCs play a crucial role in reducing oxidative stress and promoting the secretion of angiogenic factors, both of which are critical for β-cell survival and regeneration [62,63]. These findings highlight the efficacy of MSC-based therapies in treating both T1DM and T2DM, underscoring their potential as a transformative treatment option for diabetes.

Nevertheless, as discussed in the previous section, operational challenges related to cell storage and handling, along with therapeutic efficacy that varies based on donor characteristics, remain significant hurdles to the direct usage of MSCs. Furthermore, therapeutic approaches utilizing autologous MSC transplantation to avoid immune rejection are associated with impaired functionality in MSCs isolated from patients with diabetes, including defects in the capacity to regulate immune responses, multi-lineage potential, and contribution to insulin resistance [64,65]. Even upon transplantation, MSCs encounter high-glucose and inflammatory conditions, leading to low survival rates and limited biological activity [66,67]. These unresolved challenges have led to increased consideration of MSC-derived exosomes as a promising alternative. Exosomes maintain the physiological functions and low immunogenicity of MSCs while overcoming some of the inherent limitations associated with cell-based therapies. They offer advantages such as enhanced stability, ease of storage and handling, and the potential for targeted delivery of therapeutic molecules. Consequently, MSC-derived exosomes present a viable strategy for targeted tissue therapies, complementing MSC-based treatments and addressing the limitations associated with direct MSC transplantation.

## 4. MSC Exosomes and Their Use in Tissue Regeneration

MSCs possess numerous therapeutic functions, including the repair of damaged tissues owing to their self-renewal capacity, anti-inflammatory effects, multipotent differentiation, and anti-aging properties, while also exerting therapeutic effects through intercellular contact and long-term effects on target cells via the secretion of various therapeutic effectors through paracrine activity [68,69,70,71]. These regenerative properties underscore their potential for treating a variety of diseases. However, with progress in research, it has become evident that the regenerative capacity of MSCs is largely attributed to the paracrine effect mediated by the secretion of cytokines, growth factors, and chemokines [72,73] rather than to differentiation, proliferation, or fusion with damaged cells [74,75,76]. Moreover, limitations of MSC therapy, such as the potential for inducing allogenic immune rejection [77], operational challenges related to cell handling and storage, and donor age affecting the quality of functional cells [78], highlight the need for alternatives to cell-based therapies.

**Figure 1:**
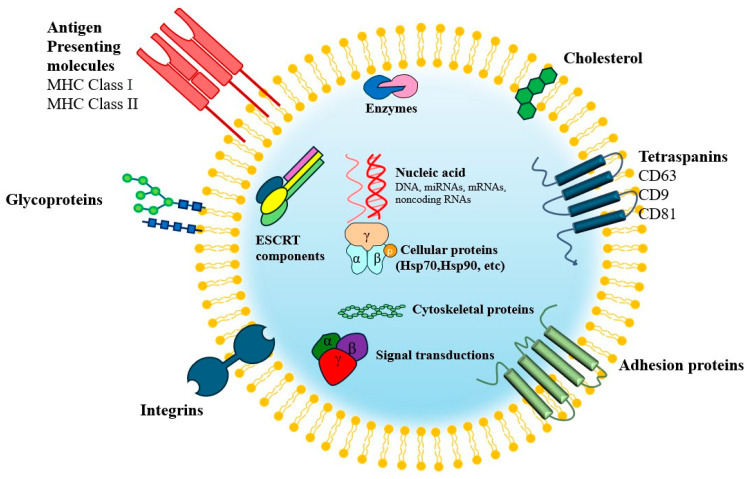
Structure of exosomes. Exosomes are extracellular vesicles secreted by cells; they contain proteins, lipids, nucleic acids, and metabolites specific to their cell of origin and are characterized by membrane proteins such as CD63, CD9, and CD81. Exosomes mediate intracellular communication and influence various aspects of cell biology. MHC class I, II = major histocompatibility complex class I, II; ESCRT = endosomal sorting complexes required for transport.

Exosomes, spherical extracellular vesicles (EVs) ranging in size from 30 to 150 nm, have emerged as a promising alternative. These vesicles are secreted by most cells and are formed through a biogenesis process involving the double invagination pathway, which includes early sorting endosomes, late sorting endosomes, multivesicular bodies, and intraluminal vesicles, although their exact formation mechanisms remain controversial [23,24] (Figure 1). Particularly, they serve as cell-mimetic carriers capable of loading the cargo of parent cells, thereby mimicking the therapeutic effects of MSCs, including tissue regeneration and immunomodulation. This highlights their potential to address the limitations of MSC-based cell therapy while preserving its advantages [31]. Specifically, in diseases with limited self-renewal and recovery capabilities, such as cartilage injuries, MSC-derived exosomes have demonstrated the ability to promote cartilage regeneration and facilitate the orderly regeneration of subchondral bone.

**Figure 2:**
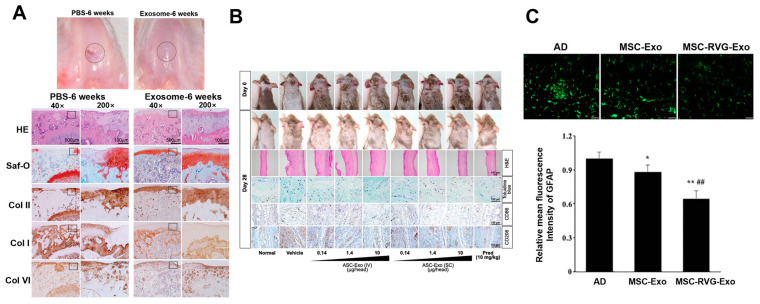
MSC exosome therapy for tissue regeneration. (**A**) At 6 weeks after surgery, superior bone regeneration and neotissue formation with good integration to host cartilage were observed in the exosome-treated group, as shown by the highlighted circle areas, in contrast to the irregular surface and structural disruptions observed in the PBS-treated control group. Histologically, the outlined box regions of the exosome-treated defects demonstrated complete neotissue filling and hyaline cartilage formation, characterized by a uniform distribution of glycosaminoglycans (GAGs) and type II collagen, with minimal type I collagen [79]. Copyright 2016 © Osteoarthritis Research Society International. (**B**) The therapeutic effect of adipose-derived stem cell (ASC)-exosomes on atopic dermatitis (AD)-like lesions in NC/Nga mice showed a dose-dependent reduction in symptoms with both intravenously (IV) and subcutaneously (SC) administered treatments, as evidenced by representative skin symptoms. Reprinted with permission [92]. Copyright 2018 © BioMed Central Ltd. (**C**) Targeted MSC-derived exosomes administered intravenously repressed astrocyte activation, as shown by representative immunofluorescence images of glial fibrillary acidic protein (GFAP) expression and quantification indicating that Alzheimer’s disease (AD) mice exhibiting the lowest GFAP levels compared to the other groups. * *p* < 0.05 and ** *p* < 0.01 vs. AD group; ## *p* < 0.01 vs. MSC-Exo group. One-way ANOVA, *n* = 5. Reprinted with permission [97]. Copyright 2019 © BioMed Central Ltd. PBS = phosphate-buffered saline; HE = hematoxylin and eosin staining; Saf-O = safranin-O; Col I, II, VI = collagen I, II, VI; RVG = rabies virus glycoprotein.

A previous study, using MSCs derived from HuES9 human embryonic stem cells, reported that cartilage repair induced by MSC exosomes administered over a defined period resembled that seen in the natural control group and showed superior effects compared to the fibrous and non-cartilaginous tissues observed in the negative control group [79] (Figure 2A). In that study, exosomes were isolated and concentrated (25× or 50×) via tangential flow filtration (TFF) using membranes with 10–1000 kDa cutoffs, followed by a final 0.2 µm filtration. This regenerative effect of MSC exosomes aligns with the findings of studies validating the cartilage repair potential of conventional MSCs [78,80]. However, some studies have noted immediate tissue complications following MSC transplantation, resulting in suboptimal recovery outcomes [81]. These findings suggest that MSC exosomes, with their low immunogenicity and cell-derived proteins (e.g., growth factors and transcription factors) and nucleic acids (DNA, mRNA, and miRNA), can complement and potentially overcome the limitations of cell-based therapies [82,83,84,85].

MSC-derived exosomes have been reported as therapeutic agents not only for tissue regeneration but also for the therapy of inflammatory diseases owing to their inherent immunosuppressive capabilities [86]. Inflammatory responses driven by pro-inflammatory cytokines and chemokines, such as interleukin (IL)-1B, IL-12, and tumor necrosis factor (TNF)-α, are primarily associated with M1 macrophages [87,88]. These responses are modulated by anti-inflammatory cytokines such as IL-4, IL-10, and transforming growth factor-β (TGF-β), which are secreted by regulatory T cells (Tregs), helper T cells, and M2 macrophages [89,90]. When inflammatory responses remain unregulated, conditions such as autoimmune diseases and inflammatory bowel diseases can arise.

Exosomes from MSCs derived from human umbilical cord Wharton’s jelly (WJMSCs) and bone marrow (BMSCs), collected through differential centrifugation steps, can induce the polarization of M1 macrophages toward M2, allowing MSC-derived exosomes to regulate inflammation and function as therapeutic agents that help maintain immune balance. Specifically, miR-223 within MSC exosomes has been shown to induce M2 polarization through increased expression of M2 markers, such as CD206, demonstrating therapeutic effects in reducing inflammation and accelerating wound healing [91]. Furthermore, adipose tissue-derived mesenchymal stem cell-derived exosomes (ASC-exosomes) reduce the infiltration of inflammatory dendritic cells (e.g., CD86+ and CD206+ cells) and the mRNA expression of inflammatory cytokines (e.g., TNF-α and IL-31) in inflammatory skin disorders such as atopic dermatitis (AD), indicating their role as inflammation modulators [92] (Figure 2B). In that study, cells and debris were removed by centrifugation and 0.22 μm filtration, and ASC-exosomes were subsequently concentrated and purified by TFF using molecular weight cutoffs of 300 or 500 kDa.

In addition to their role as inflammation modulators, MSC exosomes also show promise as drug delivery vehicles. The cell-mimetic lipid membrane composition and surface proteins of MSC exosomes contribute to their uptake by specific recipient cells, facilitating targeted delivery [93,94,95]. Building on this, through surface modifications, MSC exosomes can be engineered to improve targeting precision and minimize off-target effects, thereby enhancing therapeutic efficacy in inflammatory conditions and other diseases [96]. Binding of rabies virus glycoprotein (RVG) through DOPE-NHS coupling significantly enhances targeting of bone marrow-derived MSC exosomes obtained by sequential centrifugation steps, resulting in improved learning and memory, reduced plaque deposition, and normalized inflammatory cytokine levels in Alzheimer’s disease mice [97] (Figure 2C).

As discussed in this section, MSC-derived exosomes retain the regenerative and immunosuppressive abilities of MSCs. With their low immunogenicity and ability to be surface-engineered, MSC-derived exosomes can serve as a promising alternative to cell-based therapies, capable of targeting specific diseases. In particular, MSC exosomes are expected to offer a promising approach for elucidating disease mechanisms and potentially developing fundamental therapeutic strategies against chronic diseases with unclear pathological mechanisms, such as diabetes [98].

## 5. MSC-Derived Exosomes in the Treatment of DM

MSCs secrete exosomes carrying an array of bioactive molecules (e.g., proteins, lipids, mRNAs, and miRNAs) that can replicate many of the regenerative and immunomodulatory properties of their parent cells. Given that these nanosized vesicles can modulate various pathophysiological processes in DM, MSC-derived exosomes have garnered significant interest as promising cell-free therapeutic agents. The following subsections explore how MSC-derived exosomes exert specific effects in T1DM and T2DM, focusing on their immunomodulatory, regenerative, and insulin resistance-reversing properties.

### 5.1. Immunomodulatory Effect in T1DM

As discussed in Section 2, T1DM’s autoimmune destruction of β-cells leads to severe insulin deficiency [11]. Existing interventions (e.g., insulin injections, transplantation) face donor shortages, immune rejection, and limited replication of endogenous insulin function. Consequently, MSC-derived exosomes have emerged as a promising alternative, offering immunomodulatory and regenerative benefits.

**Figure 3:**
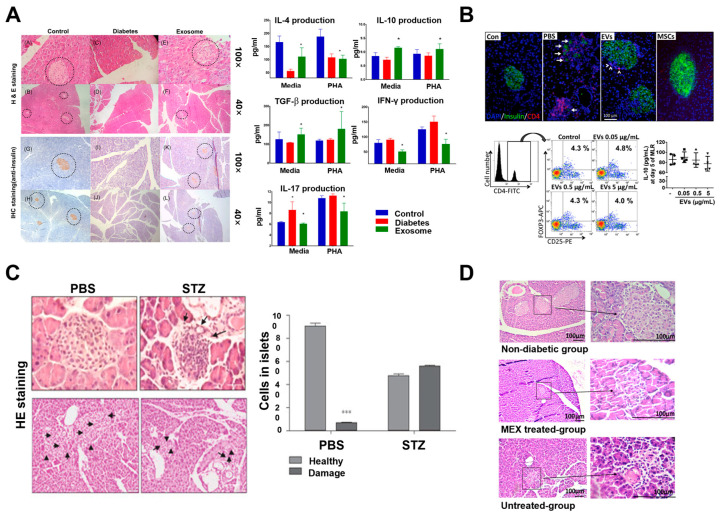
T1DM therapy using MSC-derived exosomes. (**A**) Histopathological analyses indicated that STZ-induced pancreatic islet destruction in T1DM mice was ameliorated in those treated with exosomes derived from AD-MSCs, as compared to the healthy control group. The highlighted circle regions show the restored pancreatic islets, which exhibited positive insulin staining, confirming their functionality. Treatment also increased IL-4, IL-10, and TGF-β levels while decreasing IFN-γ and IL-17 levels, demonstrating their immunomodulatory effects (* *p* < 0.05). Reprinted with permission [99]. Copyright 2018 © John Wiley & Sons. (**B**) Immunofluorescence staining showed fewer CD4+ cells in the islets of EV-treated or MSC-treated mice compared to PBS-treated mice. MSC-derived EVs suppressed T cell proliferation by inhibiting Th1 and Th17 development without inducing FOXP3+ Tregs or increasing IL-10 during the mixed lymphocyte reaction (*n* = 4). Arrows point to insulin expression, while arrowheads point to CD4 signals. Reprinted with permission [101]. Copyright 2017 © Elsevier Inc. (**C**) Histological analysis showed improved islet architecture and increased cell numbers in the MSC-Exo and MSC-treated groups, along with upregulation of amylase alpha 2A4, amylase alpha 2B, and regenerating islet-derived protein 2, compared to the PBS-treated group. Arrows indicate altered pancreatic morphology and multiple islets (*** *p* < 0.001). Reprinted with permission [102]. Copyright 2021 © John Wiley & Sons. (**D**) H&E staining of the pancreas tissue revealed new small islets in the treated groups. Immunohistochemistry showed positive insulin staining in the treated groups, and the signal was nearly absent in the untreated control group. Reprinted with permission [103]. Copyright 2019 © John Wiley & Sons. IHC staining = immunohistochemistry staining; STZ = streptozotocin; MEX = menstrual blood-derived MSC exosomes.

**Figure 4:**
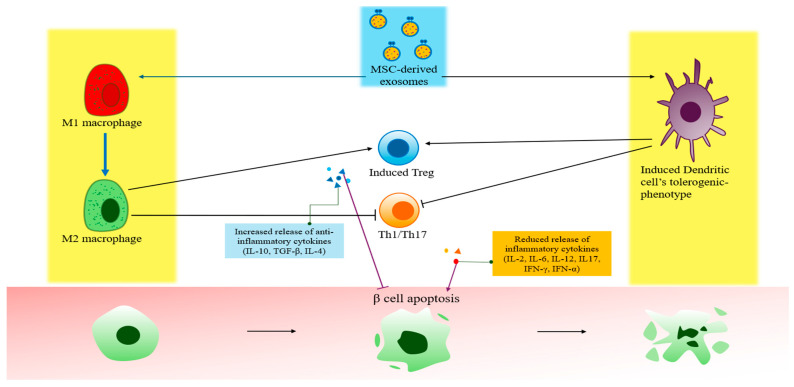
Application of exosomes for the modulation of autoimmune responses in T1DM. MSC-derived exosomes act on Mφ dendritic cells to increase Treg activity and inhibit Th1 and Th17 activity. Ultimately, MSC-derived exosomes inhibit the release of inflammatory cytokines by Th1 and Th17. This prevents β-cell apoptosis due to autoimmune responses. Th1/Th17 = type 1 T helper/T helper 17; IL = interleukin; TGF = transforming growth factor; IFN = interferon.

Adipose tissue-derived mesenchymal stem cells (AD-MSC)-derived exosomes, obtained through several ultracentrifugation steps, reportedly decrease the levels of pro-inflammatory cytokines (e.g., IFN-γ and IL-17) while increasing the levels of anti-inflammatory cytokines (e.g., IL-4, IL-10, and TGF-β) in streptozotocin (STZ)-induced T1DM rat models [99]. These cytokine changes were associated with an increase in CD25+Foxp3+ Treg cells, likely mediated by M2 macrophage polarization induced by MSC-derived exosome-mediated proliferation of mononuclear cells. This mechanism involves TGF-β and IL-10 secreted by M2 macrophages, which facilitate the conversion of native T cells into Treg cells [99] (Figure 3A). A study on bone marrow-derived mesenchymal stem cell (BM-MSC)-derived exosomes, obtained through differential ultracentrifugation, reported that dendritic cells conditioned by BM-MSC-derived exosomes transformed into immature, IL-10-secreting regulatory dendritic cells, which suppressed the priming and amplification of autoreactive T cells [100]. An ELISA analysis revealed an increased expression of immunosuppressive molecules such as IL-6, TGF-β, IL-10, and PEG2, alongside a reduction in pro-inflammatory cytokines such as IL-17 and IFN-γ, confirming the phenotype of immature dendritic cells. Co-culture of T cells with dendritic cells conditioned by BM-MSC-derived exosomes, collected via centrifugation and column resin, resulted in elevated TGF-β, IL-10, and IL-6 levels, as well as inhibition of effector T cell activation mediated by IFN-γ. The immunosuppressive effects of BM-MSC-derived exosomes were further validated by the observed increase in CD127^low^ FOXP3+ T cells and a reduction in IL-17+ cells [100]. Furthermore, approaches to addressing T1DM by enhancing the expression of immunosuppressive factors in MSCs have also been investigated [101] (Figure 3B). A previous study using MSCs with high expression of TNF-α-stimulated gene 6 (TSG-6), an immunosuppressive mediator, reported that TSG-6-high MSC-derived exosomes suppressed β-cell damage by modulating the autoimmune response. The immunosuppressive effects of TSG-6-high MSC-derived exosomes were demonstrated through the inhibition of Th1 and Th17 cell activation, as well as antigen-presenting cells, while promoting the production of the immunosuppressive cytokine IL-10 in an allogeneic mixed lymphocyte reaction assay [101] (Figure 4).

### 5.2. T1DM Inducing β-Cell Regeneration

**Figure 5:**
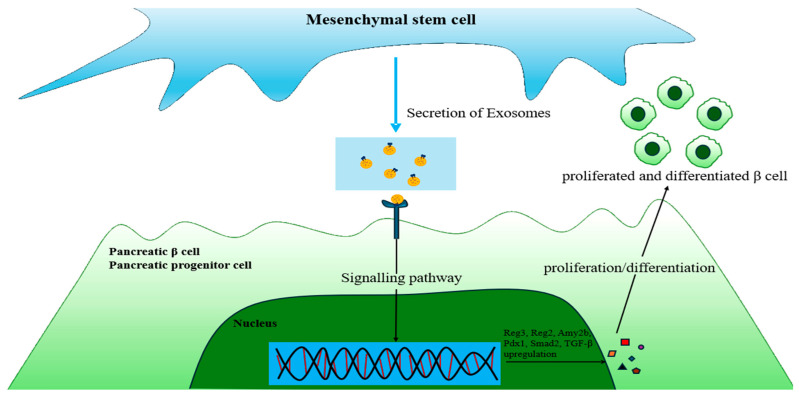
Application of exosomes for β-cell regeneration in T1DM. MSC-derived exosomes act directly on pancreatic β-cells to upregulate the expression of Reg3, Reg2, Amy2b, Pdx1, Smad2, TGF-β, and genes related to cell proliferation/differentiation. Thus, MSC-derived exosomes can regenerate damaged β-cells. Amy2b = amylase alpha 2B; PDx1 = pancreatic and duodenal homeobox 1; Smad2 = SMAD Family Member 2.

As previously noted, the autoimmune destruction of pancreatic β-cells in T1DM often leads to irreversible diabetes due to their limited regenerative capacity. Therefore, restoring β-cell function remains a key therapeutic objective. Recent studies have investigated MSC-derived exosomes to promote β-cell regeneration. For instance, a study utilizing human umbilical cord-MSC-derived exosomes (Huc-MSC-ex), enriched through differential centrifugation steps, demonstrated their ability to limit pancreatic tissue damage, promote regeneration, and enhance insulin production in STZ-induced diabetic mice, thereby improving disease outcomes [102]. Analysis of microRNA (miRNA) present in Huc-MSC-ex revealed their involvement in pathways related to cell proliferation, cell cycle regulation, inflammation, apoptosis, and metabolism. In STZ-induced diabetic mice treated with Huc-MSC-ex, a recovery in the reduced size and cell count of the islets of Langerhans was observed. Compared with the PBS-treated control group (diabetic mice injected with phosphate-buffered saline), the Huc-MSC-ex-treated group showed increased pancreatic insulin content and upregulated genes associated with insulin signaling and tissue regeneration, including amylase [102] (Figure 3C). BM-MSC-derived exosomes, enriched through differential centrifugation steps, have demonstrated therapeutic effects in T1DM [104]. In STZ-induced T1DM mice, treatment with BM-MSC-derived exosomes decreased the blood glucose levels and increased the insulin levels. Furthermore, upregulation of genes involved in pancreatic β-cell regeneration and insulin expression, including pancreatic and duodenal homeobox 1 (Pdx1), mothers against decapentaplegic homolog 2 (Smad2), mothers against decapentaplegic homolog 3 (Smad3), and TGF-β, was observed. A comparison between groups treated with BM-MSCs and BM-MSC-derived exosomes revealed more pronounced upregulation of these genes in the exosome-treated group, indicating that BM-MSC-derived exosomes may facilitate more effective β-cell regeneration and differentiation compared to cell-based therapies [104]. Similarly, menstrual blood-derived MSC exosomes (MEX), isolated using an exo-spin kit, have shown potential in promoting pancreatic β-cell regeneration in STZ-induced T1DM mice [103] (Figure 3D). Histological analysis of pancreatic tissue revealed an increase in the number and size of islets in the MEX-treated group. Pdx1 immunostaining further confirmed Pdx1 expression within islet cells, suggesting that MEX administration induced insulin production and β-cell maturation. An increase in blood insulin levels was also observed in the MEX-treated group, and the homing effect of MEX to the pancreas underscores their potential utility in T1DM therapy [103] (Figure 5).

### 5.3. Reversal of Insulin Resistance

As noted in Section 2, T2DM accounts for around 95% of diabetes cases, characterized by insulin resistance in target cells and gradual β-cell decline [14,105]. Consequently, T2DM therapies center on improving insulin sensitivity and safeguarding β-cells [14,41]. However, standard treatments (e.g., insulin injections, OHAs) provide only partial glucose control and do not fully address β-cell dysfunction [8,106].

**Figure 6:**
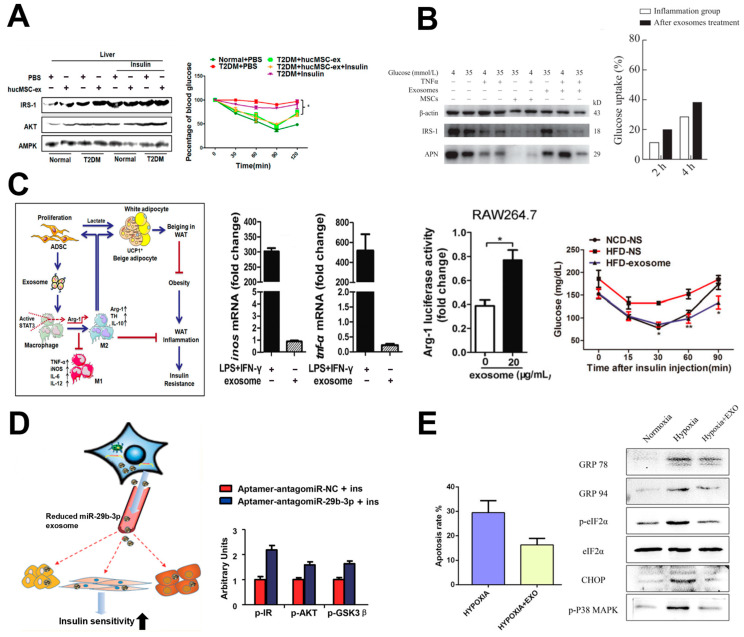
T2DM therapy using MSC-derived exosomes. (**A**) Changes in the expression of insulin signaling markers activated by Huc-MSC-ex (**left**) and insulin tolerance over time with or without Huc-MSC-ex treatment (**right**). Reprinted with permission [107]. Copyright 2018 © American Chemical Society. (**B**) Changes in the expression levels of adiponectin and IRS-1 depending on Huc-MSC-ex treatment (**left**) and the resulting effect on insulin resistance improvement (**right**). Reprinted with permission [108]. Copyright 2021 © Springer Nature. (**C**) Overview of the anti-inflammatory effects and insulin resistance improvement mechanism of ADSC-derived exosomes (**left**) reduction in inflammatory cytokines and M2 polarization effects (**center**), and the effect on insulin resistance improvement (**right**). Reprinted with permission [110]. Copyright 2017 © American Diabetes Association. (**D**) Mechanistic overview of insulin sensitivity changes in target cells according to the miR-29b-3p loading level of aged BM-MSC-derived exosomes. (**left**) and changes in insulin signaling-related protein expression upon miR-29b-3p inhibition (**right**). Reprinted with permission [111]. Copyright 2019 © American Chemical Society. (**E**) Changes in the apoptosis rate of pancreatic cells under hypoxia with or without Huc-MSC-ex treatment (**left**), and expression levels of apoptosis-related signaling proteins under hypoxia (**right**). Reprinted with permission [112]. Copyright 2020 © Springer Nature. AMPK = AMP-activated protein kinase; ARG = arginase; iNOS = inducible nitric oxide synthase; UCP1 = uncoupling protein 1; LPS = lipopolysaccharide; GSK = glycogen synthase kinase; GRP = glucose-regulated protein; eIF2 = eukaryotic initiation factor 2; CHOP = C/EBP homologous protein; MAPK = mitogen-activated protein kinase. * *p* < 0.05 and ** *p* < 0.01 vs. NCD-NS.

**Figure 7:**
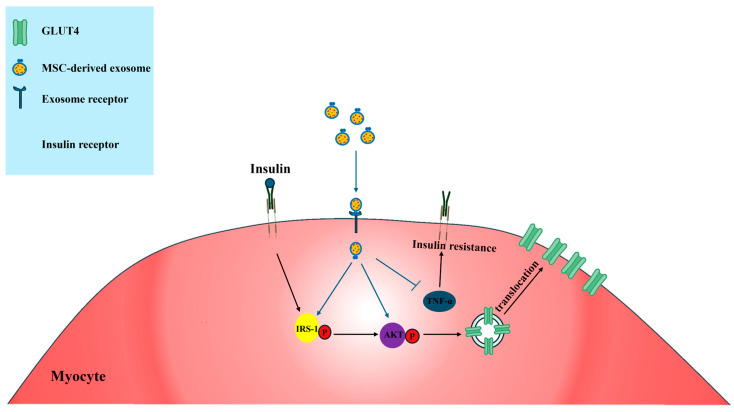
Application of exosomes for myocyte insulin resistance in T2DM. MSC-derived exosomes act on myocytes to increase the expression of p-IRS1 and p-AKT, which are involved in the insulin signaling pathway, leading to increased GLUT4 translocation. Furthermore, MSC-derived exosomes downregulate TNF-α and improve cellular insulin resistance. IRS = insulin receptor substrate. AKT = protein kinase B; GLUT = glucose transporter; Blue arrows = signal transduction process of exosome; Black arrows = signal transduction process of insulins.

Huc-MSC-ex, obtained through several centrifugation steps, have been shown to improve insulin sensitivity in peripheral tissues in high-fat diet (HFD)/STZ-induced T2DM mice [107] (Figure 6A). Intravenous administration of Huc-MSC-ex reduced blood glucose levels and promoted glucose uptake in skeletal muscle and liver cells. Huc-MSC-ex, in the presence of insulin, activates the expression of insulin signaling markers such as IRS-1 and protein kinase B (AKT), which are essential for glucose transport and metabolism, thereby attenuating the progression of insulin resistance. Furthermore, Huc-MSC-ex treatment increased the expression of the insulin-dependent glucose transporter GLUT4 in myocytes [108]. In terms of glucose metabolism, Huc-MSC-ex enhanced the expression of GSK3β, a protein involved in glycogen synthesis in hepatocytes, glycogen synthase in hepatocytes, and pyruvate kinase in muscle cells, indicating its role in regulating glucose metabolism [108,109]. Moreover, Huc-MSC-ex inhibited β-cell apoptosis caused by STZ-induced islet damage, demonstrating its β-cell protective effects [107]. Another study utilizing Huc-MSC-derived exosomes, obtained through centrifugation and concentration, demonstrated improvements in chronic inflammation and hyperglycemia, key contributors to insulin resistance in the adipose tissue of patients with T2DM [108]. In insulin-resistant adipocytes induced by TNF-α and high glucose media, Huc-MSC-ex treatment increased the expression of adiponectin and IRS-1 while reducing leptin expression (Figure 6B). Adiponectin is an adipokine that enhances insulin sensitivity and possesses anti-inflammatory properties, whereas leptin is involved in regulating energy balance and can contribute to insulin resistance when dysregulated. These findings suggest that Huc-MSC-ex may ameliorate chronic inflammation in adipose tissues and improve insulin resistance by activating IRS-1 and modulating adipokine levels [108] (Figure 7). White adipose tissue (WAT) inflammation in obese patients contributes to insulin resistance through M1 macrophage polarization, which increases the secretion of pro-inflammatory markers such as iNOS and TNF-α [110]. To address this, studies have explored the use of adipose-derived stem cell (ADSC)-derived exosomes, collected through concentration and ExoQuick-TC, to improve insulin resistance by polarizing M1 macrophages to the M2 phenotype. ADSC-derived exosomes reduce the expression of inflammatory markers such as iNOS, TNF-α, and IL-12 in LPS- and IFN-γ-stimulated macrophages. Treatment with ADSC-derived exosomes has also been shown to improve glucose tolerance and insulin sensitivity in obesity-induced mice, with the exosome-treated group exhibiting a decreased expression of M1 phenotype markers such as TNF-α, IL-12, and IL-6, and an increased expression of the M2 phenotype marker Arg-1 (Figure 6C). These findings suggest that ADSC-derived exosomes alleviate WAT inflammation and im-prove insulin resistance [110].

**Figure 8:**
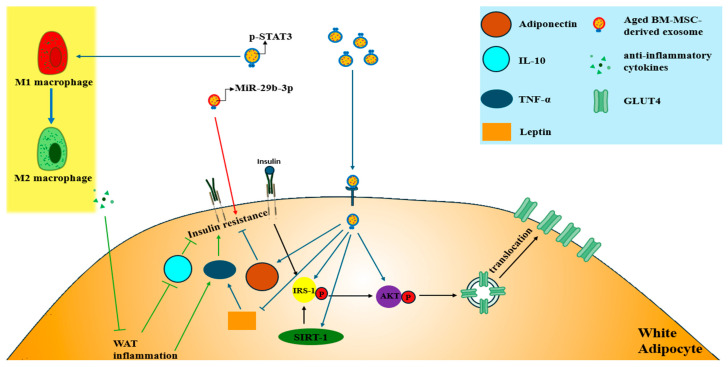
Application of exosomes for adipocyte insulin resistance in T2DM. Aged BM-MSC-derived exosomes induce insulin resistance in adipocytes. p-STAT3 present in MSC-derived exosomes acts on WAT to convert M1 Mφ to the Arg-1^high^M2 Mφ subtype. Anti-inflammatory cytokines released from the Arg-1^high^M2 Mφ subtype inhibit WAT inflammation and improve insulin resistance. MSC-derived exosomes also act on white adipocytes to increase SIRT-1 and adiponectin expression and decrease leptin expression, improving insulin resistance. STAT = signal transducer and activator of transcription; WAT = white adipose tissue; Blue arrows = signal transduction process of exosome; Red arrow = induction of IR by MiR-29b-3p in exosomes released by aged BM-MSC; Green arrows = signal transduction process of anti-inflammatory cytokines; Black arrows = signal transduction process of insulins.

Another study demonstrated that BM-MSC-derived exosomes, obtained through several centrifugation steps, from older patients with T2DM were internalized by adipocytes, myocytes, and hepatocytes, contributing to the development of insulin resistance [111]. miRNA microarray analysis revealed elevated levels of miR-29b-3p in BM-MSC-derived exosomes from aged mice compared with young mice. Upon treatment with miR-29b-3p, insulin resistance was induced in adipocytes, myocytes, and hepatocytes through binding to the 3′ UTR of SIRT1 mRNA, which suppressed SIRT1 translation [111] (Figure 6D). To address insulin resistance, BM-MSC-specific nanocomplexes/aptamer-antagomiR-29b-3p were administered to aged mice, leading to a reduction in miR-29b-3p levels in BM-MSC-derived exosomes and a subsequent improvement in insulin resistance. These findings suggest that BM-MSCs could serve as therapeutic targets for treating aging-associated T2DM [111] (Figure 8).

### 5.4. Inhibition of β-Cell Apoptosis

**Figure 9:**
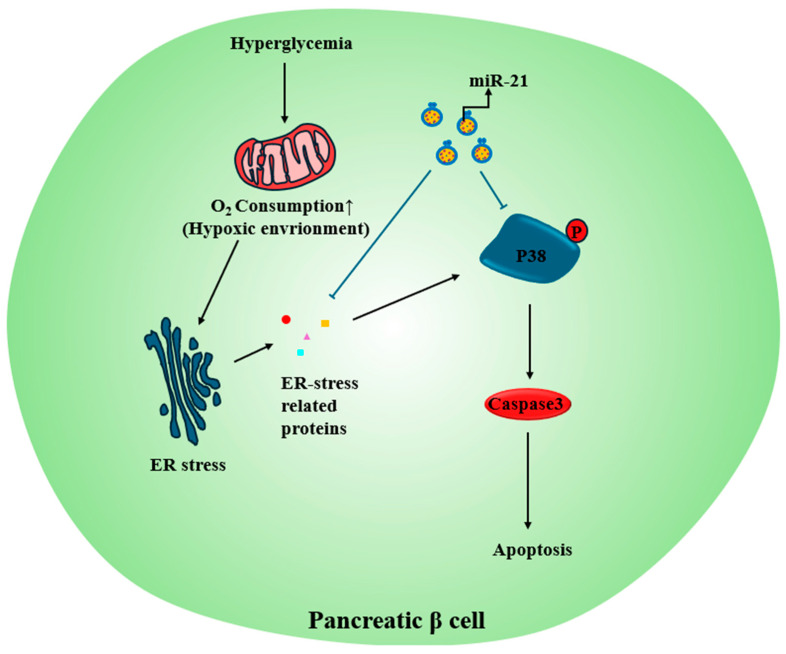
Application of exosomes for β-cell protections in a hypoxic environment. miR-21 is present in Huc-MSC-derived exosomes. It suppresses β-cell apoptosis by inhibiting ER stress-related proteins in a hypoxic environment in β-cells caused by sustained hyperglycemia. ER = endoplasmic reticulum; Blue arrows = signal transduction pathways involving miR-21 within exosomes; Black arrows = signal transduction process of hyperglycemia-induced apoptosis.

A previous section highlighted how chronic hyperglycemia in T2DM imposes metabolic stress on pancreatic β-cells. Prolonged hyperglycemia raises oxygen demand, potentially creating hypoxic conditions and triggering β-cell apoptosis [113]. To enhance β-cell survival and function, studies have investigated the use of MSC-derived exosomes. MSCs are known to release large quantities of exosomes under hypoxic conditions [114], and their application has demonstrated improved β-cell survival and regeneration [112]. Co-culture of βTC-6 cells (mouse β-cell line) with Huc-MSC-derived exosomes, isolated through several centrifugation steps and concentration, under hypoxic conditions at high concentrations (50, 100, 200 μg/mL) enhanced cell survival and reduced the expression of ER stress-related proteins, including eIF2α, CHOP, and glucose-regulated protein 78 (GRP78). Sequencing analysis of exosome contents revealed an enrichment of miRNAs such as miR-21, let-7g, miR-1246, miR-381, and miR-100. Among these, miR-21 was shown to directly inhibit cell apoptosis by suppressing the expression of eIF2α, CHOP, GRP78, and GRP94 and inhibiting the activation of the p38 MAPK signaling pathway, providing insights into the therapeutic mechanisms of Huc-MSC-ex in β-cell therapies [112] (Figure 6E and Figure 9).

Overall, MSC-derived exosomes from various sources have demonstrated significant potential in addressing both T1DM and T2DM through multiple mechanisms by combining immunomodulatory, regenerative, and metabolic regulatory functions. In T1DM, exosomes modulate immune responses by decreasing pro-inflammatory cytokines (e.g., IFN-γ and IL-17) and increasing anti-inflammatory cytokines (e.g., IL-4, IL-10, and TGF-β) in STZ-induced T1DM rat models. These cytokine changes are associated with an increase in regulatory T cells (CD25+Foxp3+ Treg) and M2 macrophage polarization, mediated by MSC-derived exosome-induced proliferation of mononuclear cells. In addition, MSC-derived exosomes transform dendritic cells into immature, immunosuppressive phenotypes, further suppressing autoreactive T cell activity and enhancing the production of immunosuppressive cytokines like IL-10. Enhanced expression of immunosuppressive factors such as TSG-6 in MSCs also contributes to the suppression of β-cell damage by modulating autoimmune responses. In T2DM, MSC-derived exosomes improve insulin sensitivity and glucose metabolism by upregulating insulin signaling pathways, increasing GLUT4 expression, and reducing insulin resistance markers. They also alleviate chronic inflammation by inducing macrophage polarization into the M2 phenotype and decreasing pro-inflammatory cytokines. Furthermore, preventing pancreatic β-cell apoptosis leads to promoting their regeneration and enhancing insulin secretion. miRNAs such as miR-21, let-7g, and miR-29b-3p in the exosomes are known to contribute to such therapeutic mechanisms.

## 6. Conclusions

Current MSC-based therapies have demonstrated potential in addressing DM by improving insulin sensitivity, modulating immune responses, and promoting tissue regeneration. MSCs alleviate inflammation in peripheral tissues and support β-cell survival under stress conditions via paracrine secretion of bioactive molecules such as cytokines, growth factors, and extracellular vesicles. These properties underscore that MSC-based therapies are valuable approaches for addressing the complex pathophysiology of diabetes. Nonetheless, several challenges, such as donor variability, difficulties in cell storage and handling, and reduced biological activity in hyperglycemic and inflammatory conditions, limit the broader application of MSC-based therapies. The low survival rates of transplanted MSCs in vivo and the risk of immune rejection further complicate their clinical use.

To address these challenges, MSC-derived exosomes have emerged as a promising cell-free therapeutic option. These exosomes retain the regenerative and immunomodulatory properties of MSCs while offering advantages such as low immunogenicity, enhanced stability, and ease of storage. Furthermore, various engineering strategies, such as surface modifications for tissue-specific targeting or drug loading to incorporate additional therapeutic agents, can enhance the therapeutic potential of MSC-derived exosomes.

However, current exosome-based therapies face several technical challenges. For instance, the quality and quantity of exosomal cargo are influenced by the MSC donor, with studies indicating that young MSC donors produce a richer secretome, leading to higher therapeutic efficacy. Additionally, achieving therapeutic doses requires large-scale in vitro cell culture platforms and advanced exosome isolation and purification techniques. Moreover, standardization is essential to facilitate clinical translation and ensure consistent outcomes.

In conclusion, MSC-derived exosomes represent a promising approach to addressing challenges in DM treatment. By regulating immune responses, protecting β-cells, and improving insulin sensitivity, they offer a comprehensive therapeutic strategy for both T1DM and T2DM. While overcoming the current limitations is essential, continued advancements in this field are expected to establish MSC-derived exosomes as a valuable tool in the development of innovative and effective DM therapies.
